# Reducing ventilator-associated pneumonia in neonatal intensive care unit using “VAP prevention Bundle”: a cohort study

**DOI:** 10.1186/s12879-015-1062-1

**Published:** 2015-08-06

**Authors:** Seham F. A. Azab, Hanan S. Sherbiny, Safaa H. Saleh, Wafaa F. Elsaeed, Mona M. Elshafiey, Ahmed G. Siam, Mohamed A. Arafa, Ashgan A. Alghobashy, Eman A. Bendary, Maha A. A. Basset, Sanaa M. Ismail, Nagwa E. Akeel, Nahla A. Elsamad, Wesam A. mokhtar, Tarek Gheith

**Affiliations:** Faculty of Medicine, Zagazig University, 18 Omar Bin Elkhattab St, Al Qawmia, Zagazig City, Al Sharqia Governorate Egypt

**Keywords:** Neonate, Ventilator-associated pneumonia, Infection

## Abstract

**Background:**

Ventilator-associated pneumonia (VAP) is a serious health care- associated infection, resulting in high morbidity and mortality. It also prolongs hospital stay and drives up hospital costs. Measures employed in preventing ventilator- associated pneumonia in developing countries are rarely reported. In this study we tried to assess the efficacy of our designed “VAP prevention bundle” in reducing VAP rate in our neonatal intensive care unit (NICU).

**Method:**

This prospective before-and-after study was conducted at university hospital NICU, all neonates who had mechanical ventilation for ≥ 48 h were eligible. VAP rates were evaluated before (phase-I) and after (phase-II) full implementation of comprehensive preventive measures specifically designed by our infection control team.

**Results:**

Of 143 mechanically ventilated neonates, 73 patients developed VAP (51 %) throughout the study period (2500 mechanical ventilation days). The rate of VAP was significantly reduced from 67.8 % (42/62) corresponding to 36.4 VAP episodes/1000 mechanical ventilation days (MV days) in phase-I to 38.2 % (31/81) corresponding to 23 VAP/1000 MV days (RR 0.565, 95 % confidence interval 0.408-0.782, p = 0.0006) after VAP prevention bundle implementation (phase-II). Parallel significant reduction in MV days/case were documented in post-intervention period (21.50 ± 7.6 days in phase-I versus 10.36 ± 5.2 days in phase-II, p = 0.000). There were a trend toward reduction in NICU length of stay (23.9 ± 10.3 versus 22.8 ± 9.6 days, p = 0.56) and overall mortality (25 % versus 17.3 %, p = 0.215) between the two phases but didn’t reach statistical significance. The commonest micro-organisms isolated throughout the study were gram-negative bacteria (63/66, 95.5 %) particularly *Klebsilla pneumonia* (55/66, 83.4 %).

**Conclusion:**

Implementation of multifaceted infection control bundle resulted in reduction of VAP rate, length of stay in our NICU.

## Background

Ventilator-associated pneumonia (VAP) is defined by the Center for Disease Control and Prevention (CDC) and National Healthcare Safety Network as new and persistent radiographic infiltrates and worsening gas exchange in infants who are ventilated for at least 48 h and who exhibit least 3 of the following criteria: temperature instability with no other recognized cause, leukopenia, change in the characteristic of respiratory secretions, respiratory distress and bradycardia or tachycardia [1]. VAP is a serious complication in neonates on mechanical ventilation and account for 6.8 - 32.2 % of health-care associated infections among neonates [2, 3]. It has a large impact on neonatal morbidity, survival, hospital costs and duration of neonatal intensive care unit (NICU) stay [3, 4]. The effect of VAP on health care costs is especially significant in developing world, whereas most studies of VAP have been conducted in developed countries [5, 6].

Prevention of VAP has been primarily achieved by the “bundle approach”; this involves the simultaneous application of several preventive strategies for all patients, often aided by tools such as checklist. In some cases there is only theoretical evidence or biologic plausibility for one or more of the elements of the bundle being effective, but application of these bundles is widely used and has been highly successful [7].

As neonates have different anatomy, physiology, underlying diseases and they undergo different invasive procedures compared with adults and older children [8], specific studies for evaluating different “VAP bundles” efficacy in preventing VAP in NICU are needed. In Egypt and other developing countries, reports on the success of VAP intervention strategies, particularly among neonates, are scarce. The aim of the present work is to assess the effectiveness of our proposed “VAP prevention bundle” in decreasing rates of neonatal VAP.

## Patients and methods

### Setting

The present study was conducted in the NICU of Children Hospital of Zagazig University, Egypt from January 2013- March 2014. Our 23 bed NICU is staffed with certified physician 24 h/day, 7 days/week with a nurse-to-patient ratio 1:3-1:4 depending on the patient acuity. Eight mechanical ventilators and 5 nasal continuous positive airway pressure (CPAP) are available at our unit. A neonatologist leads daily round on all NICU patients to review patient information and develop care plan.

### Design

This before-and-after intervention prospective study passed through the following periods; **phase-I** at which VAP rate, expressed as the number of VAP episodes per 1000 mechanical ventilator days (VAP/1000 MV days) were calculated for 6 months started at January 2013. Throughout this period we reviewed and summarized recommendations by different authors and health institutes regarding strategies for prevention of health care associated infections particularly VAP. Observations were documented by our team members regarding the most prevalent practical errors that may contribute to increased risk of VAP among our mechanically ventilated neonates. Accordingly, our “VAP preventive bundle” was tailored to stress on our common errors and include common evidence-based practices recommended by previous studies and agencies [9–15].

Three months were needed (**intermediate phase**), started at July 2013, until education and full implementation of the bundle by our health care providers were satisfactory accomplished and their adherence rate reached 86 %. During this period we performed education session to discuss evidences about the pathogenesis, risk factors, danger of VAP and its sequel. Training and re-training campaigns were performed for each VAP bundle’s item particularly hand hygiene, sterile handling of respiratory equipment, and proper timed mouth care. Finally, signed statement from each staff member acknowledging their understanding of the policy and the mandate to comply with it was taken, to ensure the connection between policy and practice.

### VAP prevention bundle

In addition to routine infection control protocol, our designed bundle was composed of:

- Head-of-bed elevation 30^0^-45^0^.
- Re-enforcement of hand hygiene practice.
- Sterile suction and handling of respiratory equipment.
- Intubation, re-intubation and endotracheal tube (ETT) suction as strictly indicated by unit protocol (document).
- Change ventilator circuit if visibly soiled or mechanically malfunctioning (document)
- Proper timed mouth care with normal saline and suction of oro-pharyngeal secretion.
- Daily evaluation for readiness for extubation to nasal continuous airway pressure (NCPAP) at morning round, and sedation vacation for sedated patient.

**Fig. 1:**
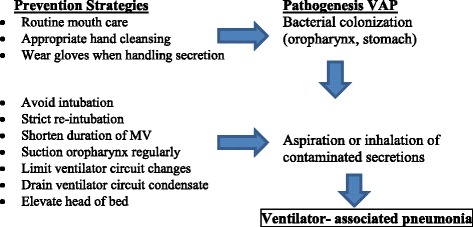
Relationship between VAP pathogenesis and its preventive strategies

Written protocols were performed for strict indications of intubation, re-intubation, suctioning of ETT and change of the ventilator circuit. Documentation is needed in the patient flow sheet. Figure 1 explains the relation between the pathogenesis of VAP and our bundle strategies.

**Phase-II** started on October 1^st^ 2013 for six months at which re-evaluation of VAP rate/1000 MV days were performed to assess the efficacy of our infection control bundle.

As ***VAP*** preventive bundle was part of our Quality Control Program where internal committee (registered nurses, infection control physicians) was assigned for continuous observation of adherence of our unit team with monthly report about this rate. ***VAP diagnosis***: VAP was diagnosed by pediatrician and confirmed by attending neonatologist using criteria for less than one year established by **Foglia and colleagues** [7]. The criteria were as follow, neonatal patients who are mechanically ventilated ≥ 48 h must have new onset and persistent abnormal chest radiograph and worsening of gas exchange (desaturations, increased oxygen requirement or increased ventilator demand), and at least three of the following: temperature instability with no other recognized cause; new onset of purulent sputum, change in character of sputum, increased respiratory secretions, or increased suctioning requirement; apnea, tachypnea, nasal flaring with retraction of the chest wall or grunting; wheezing, rales or rhonchi; cough; and bradycardia (<100 beat/min) or Tachycardia (>170 beat/min).

### Patients

All neonates admitted to NICU during phase-I and II periods and utilized mechanical ventilation for ≥ 48 h were eligible. The patient demographic, date of admission, underlying disease, duration of MV, length of NICU stay, antibiotics used, and other culture positive infection while on MV were recorded for each case.

### Methods

Complete blood count (CBC), C-reactive protein, blood culture and non- bronchoscopic broncho-alveolar lavage (NB-BAL) by passing 6f-8f sterile catheter through the endotracheal tube and wedging the airway [16] were performed for all clinically suspected VAP. The clinical microbiological laboratory analyzed the samples using Bact/Alert 3D- Biomerieux-France and provided micro-organism identification followed by antibiotic sensitivity according to the isolate using Vitek MS –Biomerieux- France. Multidrug resistant organisms need special ABX sensitivity order.

### Ethics

Informed parental consent was obtained to be eligible for enrollment into the study. The study was done according to the rules of the Local Ethics Committee of Faculty of Medicine, Zagazig University, Egypt. Our institutional review committee of ethical research approved the study.

### Statistical analysis

Categorical variables were summarized as number and percent while continuous variables were expressed as mean ± standard variation. Chi-square test and student *t*-test were used for analysis of difference for categorical and continuous variables respectively. Relative risk ratio, 95 % confidence interval, and p value were determined for VAP rates outcome. The level of significance was set at p < 0.05. SPSS statistical software version 16 was used for data presentation and analysis.

## Results

A total of 143 neonates were enrolled in the current study as 62 cases in phase-I and 81 cases in phase-II. The two groups were comparable in term of gender, birth weight, gestational age and mode of delivery as shown in Table 1.

**Table 1:** Demographic and clinical characteristics of study populations

Character	Phases		p
	I	II	
Number of ventilated neonates	62	81	
Gender (male)			
Number	43	52	
%	69.3	64.1	0.52
Gestation age (week)			
< 37 Number (%)	45 (72.5)	66 (81.5)	0.21
- 30-37	20 (32.2)	34 (41.9)	
- <30	25 (40.3)	32 (39.6)	
> 37 Number (%)	17 (27.5)	15 (18.5)	
Mean ± SD	32.18 ± 4.5	31.73 ± 4.3	0.57
Birth weight (g)			
≤ 2500 Number (%)	48 (77.4)	63 (77.7)	0.99
- < 1500	28 (45)	43 (53)	
> 2500 Number (%)	14 (22.6)	18 (22.3)	
Mean ± SD	1898 ± 954	1803 ± 1074	0.59
Mode of delivery (% C/S)	32.2	43.2	0.18
Days of mech. ventilation			
Min	5	5	
Max	51	35	
Mean ± SD	21.50 ± 7.6	10.36 ± 5.2	0.000
Length of NICU (day)			
Min	7	10	
Max	63	45	
Mean ± SD	23.87 ± 10.3	22.8 ± 9.7	0.56
Mortality Number (%)	16 (25.8)	14 (17.3)	0.22

*SD* standard deviation, *C*/*S* caesarian section, *NICU* neonatal intensive care unit

The leading cause for primary use of mechanical ventilation was prematurity and related complication (46/62 cases, 74.2 % versus 63/81, 77.8 % in phase-I and phase II respectively). Other causes such as perinatal asphyxia (6/62, 9.7 % versus 11/81, 13.6 %), respiratory causes other than RDS (4/62, 6.5 % versus 3/81, 3.8 %), congenital heart diseases (5/62, 8 % versus 4/88, 4.9 %), others (1/62, 1.6 % versus 0 %) were diagnosed as primary causes for mechanical ventilation.

2500 days of mechanical ventilation were accrued during the study periods as 1154 MV days in phase-I and 1346 MV days in phase-II. 42/62 (67.74 %) episodes of VAP were diagnosed during the pre-intervention period with a rate of 36.4 VAP/1000 MV days. Significant reduction in VAP incidence rate was observed after implementation of our VAP bundle, as 31/81(38.2 %) VAP events corresponding to 23 VAP/1000 MV days (RR 0.565, 95 % CI 0.408-0.782, Z score 3.437 p = 0.0006) were diagnosed in phase II as displayed in Table 2.

**Table 2:** Ventilator-associated pneumonia rates

Item	Phases	
	Phase I	Phase II
MV Neonate	62	81
VAP episodes	42	31
MV days	1154	1346
VAP %	67.8	38.2
VAP/1000 MV days	36.4	23
Relative Risk (RR)	0.565	
95 % C.I.	0.408-0.782	
Z score	3.437	
P	0.0006	

*MV* mechanical ventilation, *VAP* ventilator-associated pneumonia, *CI* confidence interval

Concomitant significant reduction in MV days/case was obvious in the post-intervention period when compared to pre-intervention one (21.50 ± 5.2 days in phase-I versus 10.36 ± 5.2 days in phase-II, p = 0.000). There was a trend toward reduction in NICU length of stay (23.9 ± 10.3 versus 22.8 ± 9.6 days in phase I and phase II respectively, p = 0.56) but didn’t reach statistical significance. 16/62 (25.8 %) mechanically ventilated neonates died in phase-I, 2 cases of them were related to VAP caused by multidrug resistant *klebsiella pneumoniae*, compared to 14/81 (17.3 %) in phase-II, one of them caused by polymicrobial VAP (*K. pneumoniae* and *Candida*). The difference in overall mortality rates between the two phases didn’t reach statistical significance (*X*^2^ = 1.54, p = 0.215).

73 VAP events were documented throughout the study, 90.4 % (66/73) of them revealed positive isolates on culturing their NB-BAL (37/42, 88 % in phase-I &29/31, 93.5 % in phase-II). Gram negative bacteria were the most commonly isolated micro-organisms (97.2 % versus 93.1 % in phase-I and II respectively), *klebsiella pneumoniae* was the leading causative pathogen throughout the study period. No single case of Gram-positive isolates was diagnosed in phase-I cases, compared to 6.9 % (2 cases) among those in phase-II. Fungus, namely *Candida spp.* was the single isolate from one case in phase-I, but were isolated mixed with gram negative bacteria in three cases of phase-II (10.4 %) as described in Table 3.

**Table 3:** Microbiologic features of VAP pathogens

Pathogen	Phases		Total
	Phase I	Phase II	
Positive Culture (%)	37 (88)	29 (93.5)	66 (90.4)
Gram-negative (%)	36 (97.2)	27 (93.1)	63 (95.5)
*- K-pneumoniae*	32 (86.5)	23 (79.3)	55 (83.4)
*- P-aeruginosa*	2 (5.4)	4 (13.8)	6 (9)
*- E-coli*	2 (5.4)	- (-)	2 (3)
Gram-positive (%)	- (-)	2 (6.9)	2 (3)
Fungi (%)	1 (2.7)	3^a^ (10.4)	4 (6)
MDR (%)	9 (24.3)	8 (27.6)	17 (25.8)

*K* klebsiella, *P* pseudomonas, *E* Escherichia, *MDR* multiple-drug resistant, ^a^three cases in phase-II showed combined K pneumonia and candida spp isolates

## Discussion

Advances in neonatal intensive care have improved survival among very low birth weight infants. As many of them require mechanical ventilation, VAP become a major challenge. It represents an important cause of morbidity and mortality in this high-risk population [17]. Data obtained from the current study runs in parallel with this fact as 50 % (71/134) of mechanically ventilated neonates enrolled in the study were very low birth weight (VLBW) and 77 % (111/134) were premature. Developmental immaturity in the neonatal immune system including greater permeability of the skin and mucus membrane, lower level of immunoglobulin, and decreased complement activity increases their susceptibility to hospital-acquired infection. Mechanical ventilation and other invasive treatment measures are very likely to increase risk of oro-pharyngeal or trachea-bronchial colonization with pathogenic bacteria, VAP occurs when bacterial, viral or fungal pathogens enter the sterile lower respiratory tract and lung parenchyma [18].

Several studies have shown a reduction of VAP rate after guidelines implementation into a bundle [9–15]. The power of the bundle is that it brings together several evidence-based practices that individually improve care, but when applied together, may result in an even greater improvement in the desired outcome [18]. “VAP preventive bundle” implemented in the present work was associated with statistically significant reduction in VAP rates in our NICU (36.4/1000 MV days in phase-1 versus 23/1000 MV days in phase-II, p = 0.0006). All items involved in our proposed bundle were derived from controlled trials or health institutes recommendations for adults, children or neonatal VAP prevention [9–15].

Most adult VAP prevention bundles recommend elevation of the head of a ventilated patient’s bed from 30-45 degrees to reduce the risk of aspiration of contaminated oro-pharyngeal and gastrointestinal content*.****Drakulovic and colleagues*** demonstrated that a semi-recumbent position reduced the rate of clinically suspected and microbiological confirmed VAP [19]. Only one underpowered pediatric trial presented in an abstract form has evaluated this intervention and showed no effect [20]. The logic of head-of-the bed elevation is sound, it is found in almost every VAP reduction bundle and its implementation was easy and accepted by health care providers in our work.

There is unequivocal evidence that hand hygiene is the most important infection control intervention in all health care setting, but also one of the most difficult strategy to maintain. Gram negative organisms which colonize the ETT are frequently carried on the hands of the care-givers [21, 22]. Several hand hygiene training campaigns were conducted throughout the study period, 6-steps hand washing posters were displayed on all sinks, alcohol-based hand rub solution were placed at each bedside, and in the corridor between rooms to improve compliance with hand hygiene.

Breathing circuit condensate contamination can also serve as a mechanism for the pathogenesis of VAP, the condensate that collect in the tubing should be drained away to prevent aspiration [23]. Center for Disease Control and Prevention (CDC) recommended; ensuring proper sterilization of reusable respiratory care equipment, using sterile water in humidification system, periodic drainage of condensate from the breathing circuit and hand hygiene before and after contact with respiratory equipment. CDC guidelines do not recommend changing the breathing circuit unless it is visibly soiled or mechanically malfunctioning [9]. We followed the CDC strategies regarding ventilator care in our bundle. Similarly, recent study concluded that decreasing the ventilator circuit changes from every 7 days to every 14 days has no adverse effect on the rate of VAP in NICU [24]. ***Yuan and his team*** reported that the risk factors for the development of neonatal VAP were re-intubation, frequent ETT suctioning, and the duration of mechanical ventilation [25], ***Tan and his fellows*** proved the same findings [26]. The use of non-invasive measures such as nasal CPAP and nasal prong ventilation may reduce VAP rate. In time–sequenced cohort studies, reducing days of mechanical ventilation by non-invasive respiratory support decreased VAP incidence [27, 28]. Pneumonia is less common in neonates treated with nasal CPAP when compared with those intubated on MV (1.9/1000 CPAP days versus 12.5/1000 MV days, p = 0.04) [27]. Results from the German Surveillance System for VLBW infants supported the protected value of NCPAP against pneumonia, as its incidence was 1/1000 CPAP days compared to 2.5/1000 MV days [29]. In our bundle, attending physician should assess, on daily base, the readiness of every mechanically ventilated neonate for weaning to NCPAP and every effort was done to wean them as soon as possible.

Center for Disease Control and Prevention (CDC) recommended a comprehensive oral hygiene program for mechanically ventilated patient [9]. A meta- analysis by ***Pineda and colleagues*** showed reduction in VAP among adult patients treated by decontamination with oral chlorhexidine [30]. Similar protective results were concluded by meta-analysis by ***Chlebichi and Safdar*** in which chlorhexidine rinse was used [31]. Neonates are likely at greater risk for aspiration of contaminated oral secretion, because endotracheal tubes used to ventilate them are un-cuffed [18]. As chlorhexidine gluconate is not approved for infants less than 2 months, timed mouth care with normal saline and oro-pharyngeal suction were included in our bundle.

The criteria defined by ***Foglia and his colleagues*** were used throughout the present study periods to ensure uniformity of the results. ***The CDC/NHSN*** (National Health safety Network) proposed protocol clarification in July 2013, at which leukocytosis (>15.000 WBC’s) or leucopenia (<4000 WBC’s) and shift to left (>10 % band forms) were added [32]. VAP rates has been reported from both developed and developing countries, the ***National Healthcare Safety Network*** reported that VAP rate in level III NICUs of US hospital in 2010 were in the range 0.4-1.4/1000 MV days [33]. In the ***International Nosocomial Infection Control Consortium***, the mean rate from 36 NICUs around the world between January 2004-December 2009 was 9.0/1000 MV days [34]. In the ***German Nosocomial Infection Surveillance System***, the mean VAP rate was 5.5/1000 MV days [29]. On the other hand, in 55 intensive care units of 8 developing countries between 2002-2005, the overall VAP rate was 24.1/1000 MV days ranging from 10.0-52.7/1000 MV days between units [35]. Data from Asian countries suggested an incidence rate varying from 3.5-46/1000MV days in the newborn period [36]. VAP rate in our study during the post-intervention period, 23/1000 MV days, was comparable to that in developing countries, but still significantly higher than the benchmark (1.5/1000 MV days) in developed countries. The lack of respiratory therapists, overcrowding in hospital, and relatively low nurse-to-patient ratio in our country’s NICUs may explain such disparity. In addition, conduct of rigorous hospital-acquired infection surveillance on a multicenter collaborative network level by NICUs in most developed countries is a major factor in the gap. Significant reduction in mean mechanical ventilation days/case were achieved in our neonates in the post-intervention period, an important goal especially in premature to reduce the hazards of MV in such population. However, reduction in MV days was not associated with similar reduction in length of NICU stay, a finding which is expected when dealing with neonates particularly premature, as NICU stay is dependent on several factors mainly; the baby’s gestational age, severity of underlying illness and hospital course of which, health-care associated infection is an important factor.

The overall mortality rate among our cases showed a trend toward reduction during the post-intervention period, but didn’t reach statistical significance (17.3 % in phase-II versus 25.8 % in phase-I, p = 0.215). As we did not match patient to detect adjusted attributable mortality, it is not possible to conclude that the reduction in mortality is attributable to the decrease in VAP rate.

The predominant pathogen of VAP in our study was bacteria, Gram negative bacteria outnumbered Gram positive types. Similar finding was shown in ***Yuan and colleague work*** [25] and ***Xie and team trial*** [37], while *Staphylococcus aureus* and *P. aeruginosa* were the most frequently identified pathogen in VAP in western pediatric populations [38, 39]. The difference in bacterial spectrum between ours and that reported from western countries may be due to varied practices, especially antibiotic selection. Exposure of NICU patients to different antibiotics favors selection and subsequent colonization with different pathogens that may leads to VAP. Awareness of local microbiological surveillance data on hospital-acquired infection can improve the selection of appropriate therapy. Even-though, the incidence of VAP was reduced with bundle implementation in our NICU, there was no significant difference in the incidence of multi-drug resistant organisms, probably due to resistant of health care providers to follow strict antibiotics use as advised by many infection control specialist [40].

The small sample size was one of our limitations in this study; we suggest that multicenter approaches may be necessary to attain larger sample sizes and to evaluate feasibility/cost-effectiveness. Finally, future longitudinal cohort studies are recommended to validate the current findings taking into consideration the rate or level of adherence during the program.

## Conclusion

This study provided characterization of VAP in an Egyptian NICU. It demonstrated that a bundle of infection control practices can effectively reduce the occurrence of VAP during neonatal ventilation. This “VAP prevention bundle” can be extended to other NICU in Egypt and other low-income countries.

## Abbreviations

VAP: Ventilator-associated pneumonia
NICU: Neonatal intensive care unit
VLBW: Very low birth weight
ETT: Endotracheal tube
MV: Mechanical ventilator CPAP continuous positive airway pressure
CDC: Center for disease control and prevention
